# Transcutaneous auricular Vagus Nerve Stimulation and Median Nerve Stimulation reduce acute stress in young healthy adults: a single-blind sham-controlled crossover study

**DOI:** 10.3389/fnins.2023.1213982

**Published:** 2023-09-07

**Authors:** Jesus Antonio Sanchez-Perez, Asim H. Gazi, Farhan N. Rahman, Alexis Seith, Georgia Saks, Srirakshaa Sundararaj, Rachel Erbrick, Anna B. Harrison, Christopher J. Nichols, Mihir Modak, Yekanth R. Chalumuri, Teresa K. Snow, Jin-Oh Hahn, Omer T. Inan

**Affiliations:** ^1^School of Electrical and Computer Engineering, Georgia Institute of Technology, Atlanta, GA, United States; ^2^Wallace H. Coulter Department of Biomedical Engineering, Georgia Institute of Technology, Atlanta, GA, United States; ^3^College of Sciences, Georgia Institute of Technology, Atlanta, GA, United States; ^4^Department of Bioengineering, University of Maryland, College Park, MD, United States; ^5^Department of Mechanical Engineering, University of Maryland, College Park, MD, United States; ^6^School of Biological Sciences, Georgia Institute of Technology, Atlanta, GA, United States

**Keywords:** stress, multimodal sensing, physiological signals, non-invasive, Vagus Nerve Stimulation, Median Nerve Stimulation

## Abstract

Stress is a major determinant of health and wellbeing. Conventional stress management approaches do not account for the daily-living acute changes in stress that affect quality of life. The combination of physiological monitoring and non-invasive Peripheral Nerve Stimulation (PNS) represents a promising technological approach to quantify stress-induced physiological manifestations and reduce stress during everyday life. This study aimed to evaluate the effectiveness of three well-established transcutaneous PNS modalities in reducing physiological manifestations of stress compared to a sham: auricular and cervical Vagus Nerve Stimulation (taVNS and tcVNS), and Median Nerve Stimulation (tMNS). Using a single-blind sham-controlled crossover study with four visits, we compared the stress mitigation effectiveness of taVNS, tcVNS, and tMNS, quantified through physiological markers derived from five physiological signals peripherally measured on 19 young healthy volunteers. Participants underwent three acute mental and physiological stressors while receiving stimulation. Blinding effectiveness was assessed via subjective survey. taVNS and tMNS relative to sham resulted in significant changes that suggest a reduction in sympathetic outflow following the acute stressors: Left Ventricular Ejection Time Index (LVETI) shortening (tMNS: *p* = 0.007, taVNS: *p* = 0.015) and Pre-Ejection Period (PEP)-to-LVET ratio (PEP/LVET) increase (tMNS: *p* = 0.044, taVNS: *p* = 0.029). tMNS relative to sham also reduced Pulse Pressure (PP; *p* = 0.032) and tonic EDA activity (tonicMean; *p* = 0.025). The nonsignificant blinding survey results suggest these effects were not influenced by placebo. taVNS and tMNS effectively reduced stress-induced sympathetic arousal in wearable-compatible physiological signals, motivating their future use in novel personalized stress therapies to improve quality of life.

## 1. Introduction

Stress is associated with physiological and behavioral responses that are major determinants of mood, behavior, and health (de Kloet et al., 2005; Schneiderman et al., 2005). Sustained stress can increase the risk of illness, including cardiovascular (CV) disease and mental disorders (Hammen, 2005; Esler, 2017). Managing stress in daily life, however, is a challenging task given the dynamic relationship between individuals and their perceived environment. Conventional clinician support (e.g., weekly therapy sessions) and subjective assessments do not account for many of the acute changes in stress that can have detrimental effects on quality of life (Garfin et al., 2018). Hence, technologies that enable continuous stress monitoring and timely mitigation could lead to novel therapeutic paradigms and improve mental health.

One promising technological approach is the combination of physiological monitoring and Peripheral Nerve Stimulation (PNS) to sense and reduce stress during everyday life. Stress-induced changes in autonomic nervous system activity lead to widespread downstream effects, which can be quantified via physiological markers (*physiomarkers*). These physiomarkers can be derived from biosignals such as the Electrocardiogram (ECG), Electrodermal Activity (EDA), Respiratory effort (RSP), Blood Pressure (BP), Seismocardiogram (SCG), and Photoplethysmogram (PPG; Giannakakis et al., 2019; Gurel et al., 2020a; Gazi et al., 2021a). To reduce stress, PNS approaches have been shown to modulate neural pathways responsible for autonomic control. A PNS modality that has received considerable attention is transcutaneous Vagus Nerve Stimulation, wherein the auricular (taVNS) or cervical (tcVNS) vagal fibers are the stimulation targets (Yap et al., 2020; Farmer et al., 2021). We recently demonstrated that tcVNS reduced traumatic stress in several markers derived from ECG, RSP, BP, SCG, PPG, and EDA signals (Gazi et al., 2020, 2021b,c; Gurel et al., 2020a,b); manifestations of opioid withdrawal (Gazi et al., 2022a); and modulates stress-relevant limbic regions (Wittbrodt et al., 2020, 2021). taVNS has been similarly shown to effectively modulate vagal afferents (Nonis et al., 2017; Badran et al., 2018b; Yakunina et al., 2018), demonstrating promising results in neonate opioid withdrawal (Jenkins et al., 2021), Long COVID mental burden (Badran et al., 2022), and depression-related neural activity (Dietrich et al., 2008). While not studied as extensively as VNS, tMNS is another PNS modality recently shown to reduce BP in hypertensive participants (Bang et al., 2018). The hypothesized afferent mechanism of tMNS involves inhibition of sympathetic tone through C-fiber activation.

Despite the promising body of evidence supporting these PNS modalities, their relative effectiveness in reducing stress has not been evaluated. On one hand, the heterogeneity in populations, stimulation devices, and conditions in which they have been individually tested to date precludes a meta-analytic comparison. On the other hand, while the utilization of a comprehensive set of physiological signals have proven to be effective in quantifying tcVNS-induced responses, the effects of taVNS and tMNS on several peripheral physiomarkers of stress remain unknown.

In this paper, we address these gaps by studying the effectiveness of tcVNS, taVNS, and tMNS in reducing physiological manifestations of stress. We hypothesized that these active PNS modalities, compared to a sham stimulation, would induce physiomarker changes associated with reductions in stress. To test this hypothesis, we conducted a sham-controlled single-blind crossover study with young healthy participants and measured their peripheral physiological responses to acute stressors with a comprehensive set of physiomarkers derived from cardiac, vascular, and sudomotor signals. We further compared these physiomarkers' responses across all PNS sites and elucidated their effectiveness in mitigating stress. By evaluating wearable-compatible physiological signals and PNS modalities, this work may provide the basis for future wearable-based therapeutics that can provide continuous, non-invasive, and personalized neuromodulation to reduce the burden of stress.

## 2. Materials and methods

### 2.1. Human subjects study

A human subjects study was carried out under the approval of the Georgia Institute of Technology Institutional Review Board (H18452) and the Navy Human Research Protection Office (HRPO) between June 2021 and November 2022. Eligible participants were young adults with no known prior history of, and that were not taking medications for, a heart condition or neuropsychiatric disorder. The eligibility criteria further required that participants were not familiar with neuroanatomy and neuromodulation. A detailed description of the study was provided before obtaining written consent and starting the protocol. The participants were instructed to refrain from any stimulant use (e.g., caffeine) prior to the study.

### 2.2. Study protocol

**Figure 1:**
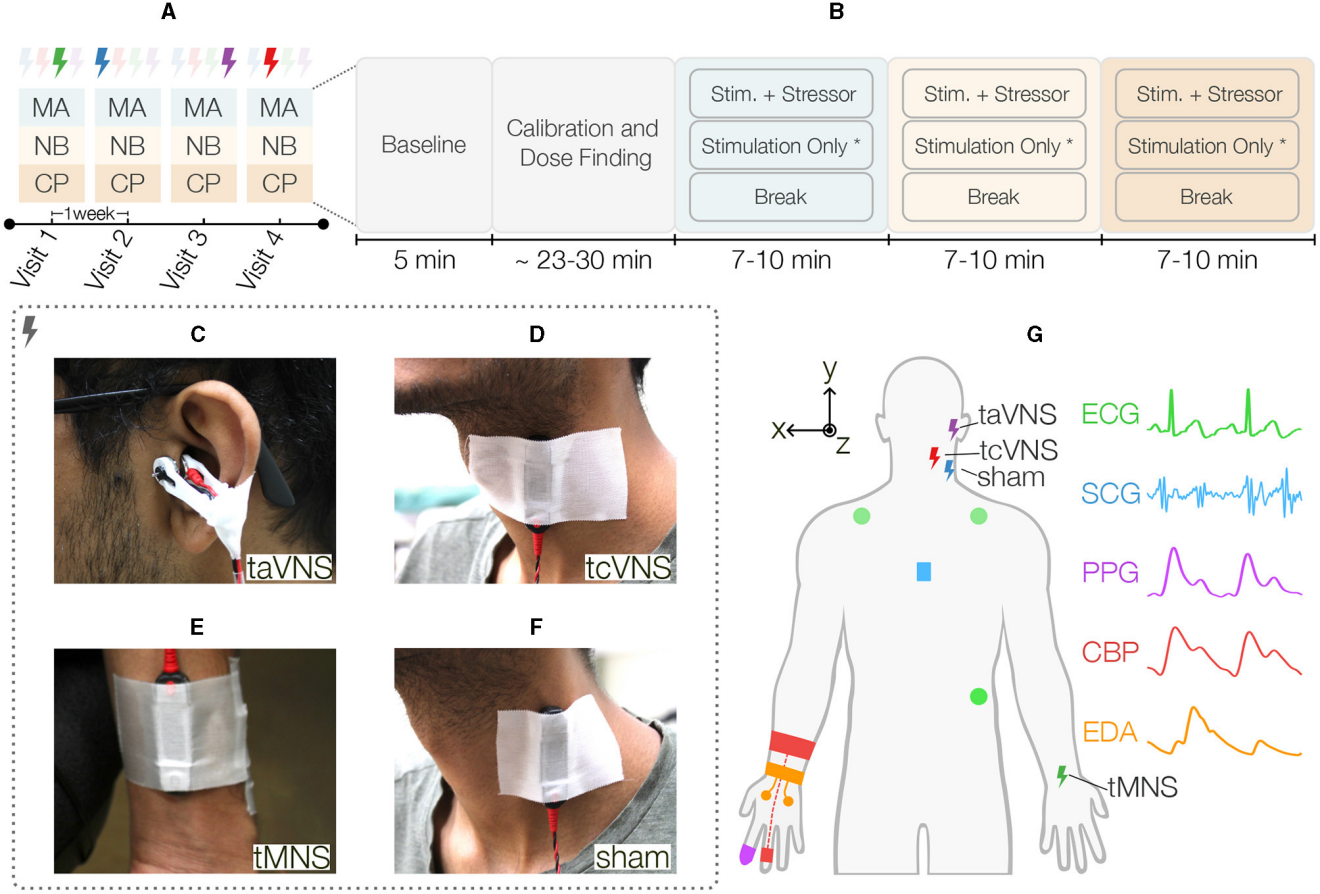
Overview of the single-blind sham-controlled crossover study. **(A)** Each participant was randomly allocated to one sequence of four different stimulation types, thereby defining the stimulation for each visit. A washout period of 1 week was used between visits. During each visit, the participants received stimulation while undergoing the following three acute stressors listed in the order they occurred in the protocol: mental arithmetic (MA), n-back (NB), and the cold pressor (CP) test. **(B)** These stressors were preceded by a rest period to capture each participant's baseline state and a period of stimulation calibration followed by a dose finding activity. Next, the stressors started in the aforementioned order, each having three distinct tasks. First, the participants underwent 2 min of the acute stressor (i.e., MA, NB, or CP) while simultaneously receiving stimulation at PT. Next, the stimulation remained active at the same intensity for another 3 min^*^. Finally, the participants went through a 5-min break period prior to starting the next stressor. The three Peripheral Nerve Stimulation (PNS) modalities evaluated in this work were **(C)** transcutaneous auricular Vagus Nerve Stimulation (taVNS), wherein a custom earclip was used to stimulate the tragus, **(D)** transcutaneous cervical Vagus Nerve Stimulation (tcVNS) that was delivered to the left side of the neck using an electrode bar placed over the carotid sheath, and **(E)** transcutaneous Median Nerve Stimulation (tMNS), which was delivered to the anterior left wrist using an electrode bar. **(F)** Sham stimulation was delivered to the left sternocleidomastoid muscle using an electrode bar. **(G)** Illustration of all PNS and physiological sensing modalities employed in this work. Five physiological signals were measured peripherally: three-electrode Electrocardiogram (ECG) in Lead II configuration, Seismocardiogram (SCG) captured at the mid-sternum with a custom accelerometer module, finger-clip based Photoplethysmogram (PPG), continuous Blood Pressure (CBP) measured with a finger cuff embedded with a blood volume pulse sensor, and electrodermal activity (EDA) measured at the hand palm with two electrodes. ^*^The stimulation-only period was not followed for tcVNS, thereby resulting in stressors' durations ranging between 7 and 10 min.

To evaluate all three PNS sites against a sham, this work employed a single-blind cross-over study design with four periods (hereafter “visits”) and various sequences formed from the four stimulations. A detailed description of the study is shown in Figure 1. Each participant was randomly allocated to one of the sequences, thereby defining the stimulation sequence to be followed during their four visits (Figure 1A). These sequences were generated by drawing multiple four-digit numbers from a random number generator, wherein each digit took values between 1 and 4 and no digits' repetition was allowed. A washout period of 1 week was used between visits. During each visit, the participants received stimulation while undergoing the following three acute stressors listed in the order they occurred in the protocol: mental arithmetic (MA), n-back (NB), and the cold pressor (CP) test. During MA, subjects were asked to repeatedly add the digits of a three-digit number and then add the result to the original number (Al'Absi et al., 1997; Gurel et al., 2019). For NB, a sequence of digits (0–9) were presented on the screen one-by-one and the subjects had to respond the number shown two-digits back (Callicott, 1999). A custom graphical user interface with voice recognition capability was used to automatically display mathematical prompts and save the responses. The CP test consisted of submerging the left foot in an ice bucket (Lovallo, 1975; Allen et al., 1987).

As shown in Figure 1B, these stressors were preceded by a 5-min rest period to capture each participant's baseline state and a period of stimulation calibration followed by a dose finding activity, both lasting ~23–30 min altogether. The calibration period was used to find each stimulation's perceptual threshold (PT). Therein, the simulation intensity was increased from zero to the maximum level that was tolerable and safe for the participant, which was defined as 150% PT. PT was then computed as 66.7% of this value. The dose finding portion measured the physiological responses at 50, 100, and 150% of this value. Next, the stressors started in the aforementioned order, each having three distinct tasks. First, the participants underwent 2 min of the acute stressor (i.e., MA, NB, or CP) while simultaneously receiving stimulation at PT. Next, the stimulation remained active at the same intensity for another 3 min. This stimulation-only period was not followed for tcVNS to not exceed the 2 min of stimulation time commonly reported in the literature for this site (Redgrave et al., 2018; Yap et al., 2020). Finally, the participants went through a 5-min break period prior to starting the next stressor. A blinding survey was asked at the end of the protocol to assess blinding effectiveness. All participants were seated throughout the study, which took place in a controlled laboratory room with air conditioning set to a temperature of 73° Fahrenheit.

### 2.3. Peripheral nerve stimulation

**Figure 2:**
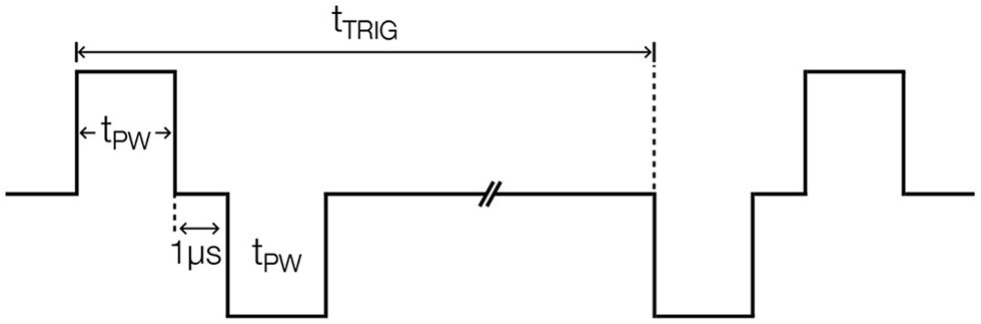
Peripheral nerve stimulation waveform with annotated parameters.

The DS8R current stimulator (Digitimer, Broadway, Letchworth Garden City, UK) was used to deliver all four stimulation types. The stimulation waveform parameters were configured with a trigger signal externally provided. As illustrated in Figure 2, the stimulator delivered a pulsed 1-cycle square alternating symmetrical bi-phasic waveform with a fixed inter-phase interval of 1 μ*s* and two stimulation-specific parameters: pulse width (*t*_*PW*_) and trigger duration (*t*_*TRIG*_). Conductive gel was used to reduce skin-electrode impedance.

*taVNS* stimulation (*t*_*TRIG*_: 40 ms, *t*_*PW*_: 500 μs; Badran et al., 2019) was delivered via custom developed earclip attached to the left tragus and secured with surgical tape (Figure 1C). The earclip design housed a pair of 10 mm disc stimulating electrodes (Digitimer) that delivered electrical stimulation across the tragus, with the anode on the inner side (Badran et al., 2018a).

*tcVNS* stimulation (*t*_*TRIG*_: 40 ms, *t*_*PW*_: 100 μs; Gurel et al., 2020a) was delivered with an electrode bar containing two round 10 mm electrode-surfaces spaced by 30 mm (Digitimer). The bar was attached to the left side of the neck over the carotid artery using surgical tape, with the anode at the bottom (Figure 1D). A hook-and-loop strap was used to maintain the bar pressed against the skin (Gazi et al., 2022a).

*tMNS* stimulation (*t*_*TRIG*_: 100 ms, *t*_*PW*_: 200 μs; Bang et al., 2018) was delivered to the anterior left wrist between the palmaris longus tendon and the flexor carpi radialis (Bang et al., 2018), using the aforementioned electrode bar with the anode toward the body (Figure 1F).

*sham* stimulation (*t*_*TRIG*_: 100 ms, *t*_*PW*_: 200μs) was delivered to the left neck using the same electrode bar placed over the sternocleidomastoid muscle, as in Lerman et al. (2019), with the anode at the bottom (Figure 1E).

### 2.4. Stimulation blinding

A single-blind process was enforced by only revealing the actual stimulation delivered and the stimulation sequence allocated for each subject to the research staff. The proportion of active sites was similarly not revealed to the subjects. At the end of the protocol, the blinding effectiveness was evaluated with the following survey question “What type of stimulation do you think you received? median, cervical vagal, auricular vagal, or sternocleidomastoid,” to which the participants responded with one of the options.

### 2.5. Physiological monitoring

As illustrated in Figure 1G, this work employs five non-invasive physiological signals continuously measured throughout the protocol: ECG, SCG, PPG, continuous BP (CBP), and EDA. The ECG signal was measured using three Ag/AgCl gel electrodes in Lead II configuration. The Biopac RSPEC-R wireless module (Biopac Systems, Goleta, CA) was used to measure and amplify ECG signal. The PPG signal was sensed at the right index finger with a transmissive finger clip PPG transducer (BST09001S, Shanghai Berry Electronic Tech, Shanghai, China) and interfaced to a wired amplifier (PPG100C, Biopac Systems, Goleta, CA). The CBP signal was measured using a finger cuff embedded with a blood volume pulse sensor on the ring finger of the right hand (ccNexfin, Edwards Lifesciences, Irvine, CA, USA). Further, a continuous EDA signal was recorded at the right hand using the E4 wristband (Empatica, Cambridge, MA). Two Ag/AgCl electrodes connected to the E4 via snap electrode cables were used to measure the EDA at the palm.

The SCG signal was measured with a custom accelerometer module attached to the anterior chest, at the mid-sternum level, with surgical tape. This module was wired to a custom Analog Front-End (AFE) that filtered and amplified the tri-axial acceleration signals (Supplementary Figure 1). The three AFE outputs (Acc_*x, y, z*_) were then interfaced to a wired data acquisition system (MP-150, Biopac Systems, Goleta, CA) that simultaneously acquired all signals described herein at a sampling rate of 2 kHz. In this work, the SCG signal was defined as the acceleration in the dorsoventral axis (Acc_*z*_).

### 2.6. Signal processing and physiomarker extraction

The continuous physiological signals were processed in MATLAB R2020b (MathWorks, Natick, MA) to extract a comprehensive set of cardiovascular and sudomotor physiomarkers, leveraging published and custom processing pipelines. The parameters of published methodology remained unchanged, unless otherwise specified.

#### 2.6.1. ECG

After filtering the ECG signals and assessing their quality, a set of “clean” (i.e., good quality) normal-to-normal intervals were extracted and used to compute instantaneous Heart Rate (HR) values following prior work's methodology (Gazi et al., 2022b). These intervals were then used to index the remaining beat-wise signals in a beat-by-beat fashion.

#### 2.6.2. PPG

PPG signals were filtered, beat-segmented, and quality assessed prior to extract the Pulse Arrival Time (PAT) and its amplitude (PPGamp; Gazi et al., 2021b).

#### 2.6.3. CBP

CBP signals were first filtered using a lowpass filter with cut-off frequency of 8 Hz, designed with the Parks-McClellan method (Parks and McClellan, 1972). Following beat-segmentation, a “clean” set of CBP beats was then created through a processing procedure that resembled PPG processing in prior work (Gazi et al., 2021b), but with a modified Signal Quality Index (SQI) procedure. Specifically, beat quality was assessed leveraging the *jSQI* function from the PhysioNet Cardiovascular Signal Toolbox (Vest et al., 2018). The following markers were then computed from this final set of beats using PhysioNet: Diastolic Blood Pressure (DBP), Systolic Blood Pressure (SBP), and Pulse Pressure (PP). The Mean Arterial Pressure (MAP) was computed using trapezoidal numerical integration.

#### 2.6.4. SCG

A semi-automatic version of our prior SCG processing methods was implemented in this work (Gazi et al., 2021b). Following filtering and beat-segmentation, a two-dimensional (2D) gray-scale image was created by vertically stacking the chopped SCG beats to facilitate the visual tracking of peaks and valleys, as well as noisy segments. The process then involved re-initializing the core peak tracking algorithm (Zia et al., 2019) utilized therein as needed to ensure correct tracking of the Aortic Opening (AO) and Aortic Closing (AC) fiducial points throughout the recording. The Pre-ejection Period (PEP) was computed as the time difference between AO and the corresponding indexing ECG R-peak (in seconds) and the Left-Ventricular Ejection Time (LVET) as the time difference between the AC and AO points. After removing outliers following prior work (Gazi et al., 2021b), the ratio of PEP to LVET (PEP/LVET) was obtained and the Pulse Transit Time (PTT) was computed by subtracting PEP from PAT (Gazi et al., 2021b). Finally, a HR-corrected LVET index (LVETI) was calculated following the general form of the widely-used Weissler's correction equations (Weissler et al., 1968; Lewis et al., 1977). See Supplementary material for detailed steps.

#### 2.6.5. EDA

The EDA signals were first assessed for quality using the methodology described in Kleckner et al. (2018). The signal was then decomposed into the tonic, phasic, and sparse components using the cvxEDA convex optimizer (Greco et al., 2016a,b). The latter two components were used to extract the EDA orienting responses (OR) and tonic statistical features, respectively (Ihmig et al., 2020; Gazi et al., 2021a). The OR were identified from the peaks in the sparse component. The number of OR (nOR) and mean magnitude of OR (mmOR) features were then computed by counting the peaks and averaging their heights, respectively, within a 10-s non-overlapping window rolled over the entire OR sequence (Gazi et al., 2021a). Three features were finally extracted from the EDA tonic component using the same windowing parameters, namely the mean (tonicMean), standard deviation (tonicSD), and normalized first differences (tonicNFD; Ihmig et al., 2020). See Supplementary material for detailed steps.

### 2.7. Feature time series generation

All feature time series were linearly re-sampled to 1 Hz and smoothed using a moving average filter with a window length of 5-s and 80% overlap. This final processing step was carried out to focus the analysis on the physiological markers' trends. The resulting feature time series were then summarized (i.e., averaged) into 10 bins, corresponding to the baseline and the three protocol segments for each of the three stressors (Section 2.2). Specifically, the “BSL” segment was defined as the first 30 s of the initial baseline period. For each stressor (MA, NB, CP), the segments where the subjects were undergoing both the stimulation and the stressor (“STM+STR”) and only the stimulation (“STM-ON”) were summarized using the second 60-s of data to allow the markers to respond across all subjects. The remaining three bins corresponded to the break periods (“BRK”) for each stressor, which were defined as the first two minutes. This definition was motivated by the observation that some subjects' physiomarkers started reacting before the stressors' onset. Finally, all markers were expressed as percentage change from any given session's baseline, except HR, BP, and the EDA features that were expressed as changes from baseline in their respective measurement units. Features were expressed this way to streamline interpretation in the case of HR and BP, and to prevent the small values in the EDA features to drive the percentage changes out of scale. The dose finding data were not analyzed in this work, as the focus here was on the comparison between the different stimulation sites and sham for a fixed stimulation level (i.e., PT).

### 2.8. Statistical analysis

To assess the stimulation effects on the stress responses of each physiomarker, linear mixed-effects (LME) models were fit to the data at each timepoint (Bates et al., 2015). These models included the normalized-to-baseline marker as the response variable and the stimulation as the fixed effect of interest. The visit and stimulation-by-visit terms were also included as fixed effects to adjust for period and carry-over effects, respectively. Type III tests were conducted to obtain estimates of the effects and the *p*-values were obtained with Satterthwaite's degrees-of-freedom approximation method (Kuznetsova et al., 2017). Significant stimulation effects were followed-up with pairwise comparisons, and the p-values were correspondingly corrected with Tukey confidence level adjustment. Timepoints with simultaneous interaction or visit effects were not studied further given the inability to elucidate the stimulation effects in isolation from other confounders. A two-tailed *p*-value < 0.05 was considered significant in this work. LME models were fit on standardized data to streamline interpretation. Linear model assumptions were verified with residual and quantile-quantile (qq) plots.

The blinding survey responses were compiled for each visit and compared against the actual stimulation type using 4 × 4 contingency tables. The Cohen's unweighted Kappa statistic between the actual and observed responses was then computed for each visit to assess blinding effectiveness. Chi-square tests of independence were also performed in each visit to assess the mutual dependency of the responses and the actual stimulation types. All statistical analysis and data visualization were carried out in R (R Core Team, 2022). Data summaries are shown in terms of the mean (μ) and standard error of the mean (*SE*), unless stated otherwise.

## 3. Results

### 3.1. Participants characteristics

A total of 24 subjects were recruited and provided written consent in this study. Five subjects dropped out of the study and their data was discarded, including one related to possible side effects of stimulation (Supplementary material). Three participants that missed the last visit were included in the final dataset due to having completed their sham session. The resulting sample size for each stimulation was 19, 17, 19, 18 for sham, tcVNS, tMNS, and taVNS, respectively. The final cohort consisted of 10 females and 9 males (age: 21 ± 2 years, height: 169 ± 11 cm, weight: 64 ± 12 kg; expressed as μ±sd). The calibration procedure resulted in the following PTs 2.52 ± 0.14, 4.51 ± 0.41, 3.75 ± 0.23, and 1.57±0.40 mA for sham, tcVNS, tMNS, and taVNS, respectively.

### 3.2. Blinding effectiveness

The Cohen's κ statistic results for every visit are shown in Table 1 and the corresponding contingency tables are shown in Supplementary Table 4. All κ values were below 0.1. In the Chi-square tests (Supplementary Table 5), no significant association between the actual and surveyed stimulation types existed on any visit.

**Table 1:** Blinding survey agreement between actual and surveyed stimulation allocations.

	**Visit 1**	**Visit 2**	**Visit 3**	**Visit 4**
Cohen's **κ**	0.0886	0.0389	−0.0556	−0.0608

### 3.3. PNS-induced changes in physiological manifestations of stress

**Figure 3:**
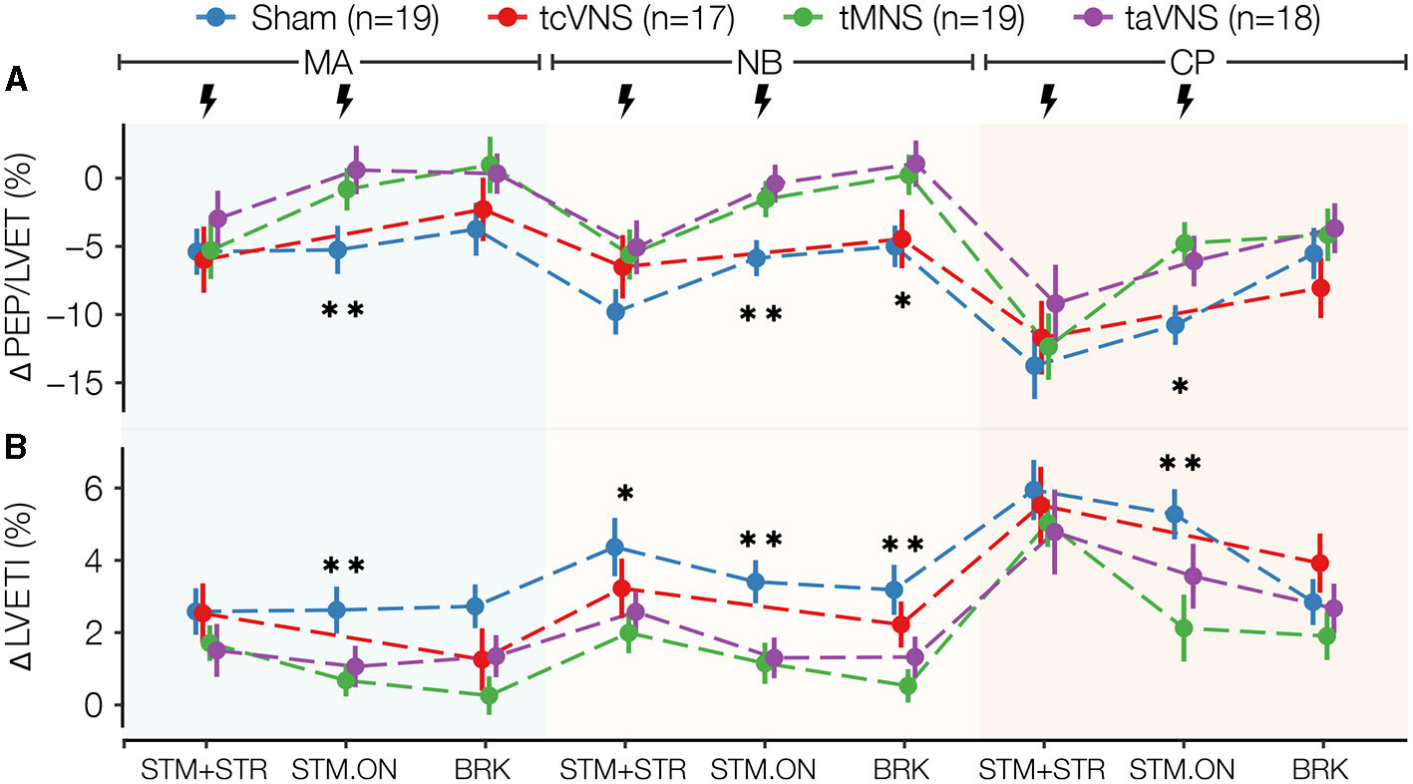
Selected cardiac stress physiomarkers time series for all stimulations: **(A)** ΔPEP/LVET and **(B)** ΔLVETI. Data points are expressed as μ±*SE*. Segments with significant main stimulation effects are identified with stars (^*^p < 0.05 and ^**^p < 0.01).

**Figure 4:**
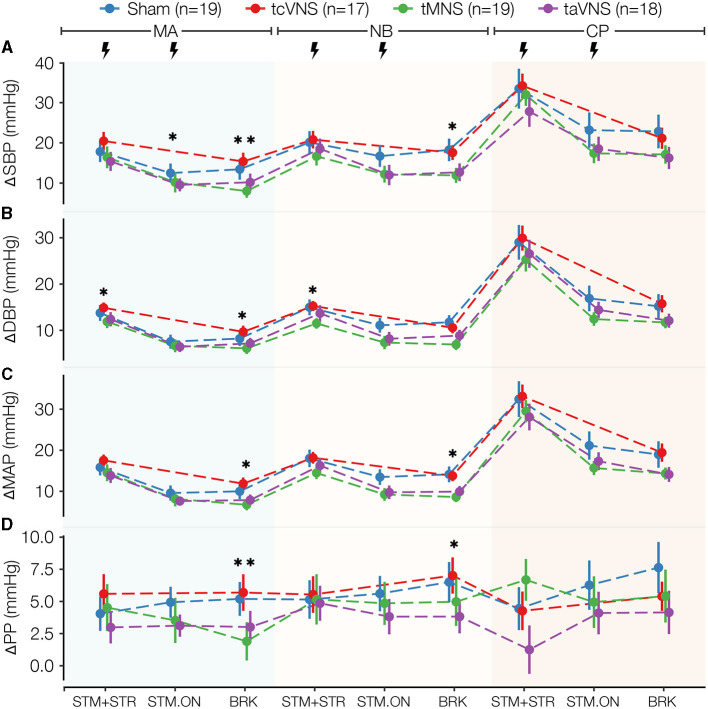
Blood pressure stress physiomarkers time series for all stimulations: **(A)** ΔSBP and **(B)** ΔDBP, **(C)** ΔMAP, **(D)** ΔPP. Data points are expressed as μ±*SE*. Segments with significant main stimulation effects are identified with stars (^*^p < 0.05 and ^**^p < 0.01).

**Figure 5:**
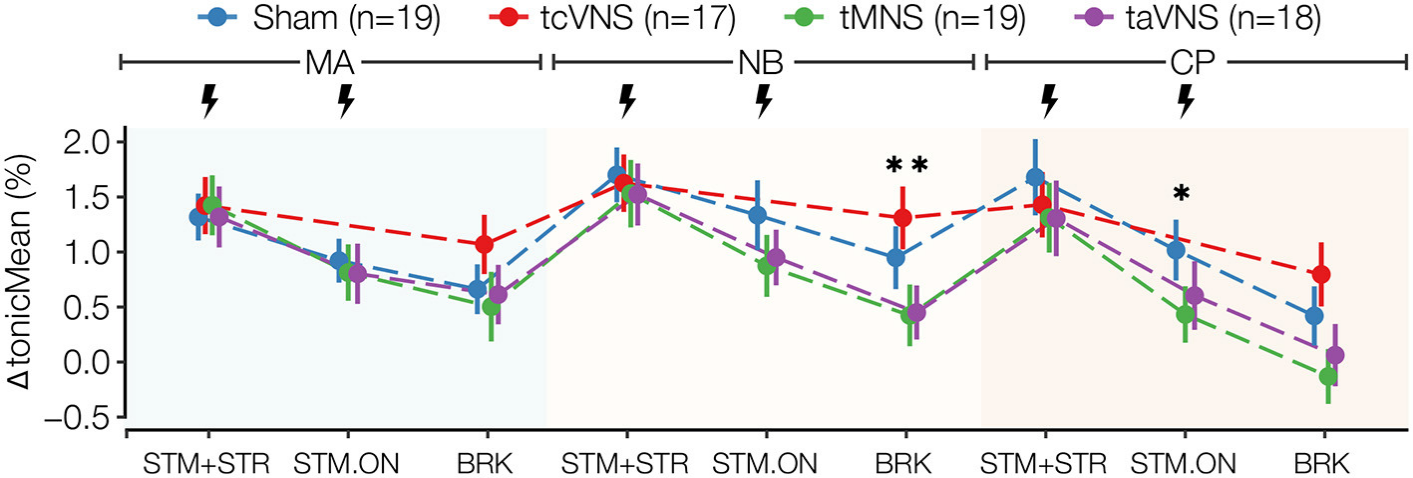
ΔtonicMean stress physiomarker time series for all stimulations. Data points are expressed as μ±*SE*. Segments with significant main stimulation effects are identified with stars (^*^p < 0.05 and ^**^p < 0.01).

Supplementary Figure 2 illustrates an exemplary SCG annotation result. The physiological responses for ΔPEP/LVET, ΔLVETI, ΔSBP, ΔDBP, ΔMAP, ΔPP, and ΔtonicMean are shown in Figures 3–5. The physiological responses for ΔHR, ΔPEP, and ΔPPGAmp are shown in Supplementary Figure 3. Supplementary Table 1 shows significant main stimulation effects resulting from the LME models at each timepoint and the corresponding *post-hoc* results are shown in Supplementary Table 2. The physiological responses μ±*SE* summaries for each segment are shown in Supplementary Table 3.

As illustrated in Figure 3, PNS induced significant stimulation effects on the cardiac markers ΔPEP/LVET and ΔLVETI (Supplementary Table 1). Compared to sham, taVNS and tMNS similarly lowered ΔLVETI and increased ΔPEP/LVET responses during all timepoints studied. tcVNS yielded similar trends, but the responses were closer to sham. Specifically, the *post-hoc* tests revealed that taVNS significantly increased ΔPEP/LVET compared to sham by 5.9, 5.5, and 6.0% in MA, NB, and CP STM-ON, respectively (Supplementary Tables 2, 3). For ΔLVETI, tMNS was found to significantly decrease the response compared to sham by 1.9% in MA-STM-ON, 2.2% in NB-STM-ON, 2.7% in NB-BRK, and 3.2% in CP-STM-ON (Supplementary Tables 2, 3). Meanwhile, taVNS was found to significantly reduce ΔLVETI compared to sham by 1.5% in MA-STM-ON and 2.1% in NB-STM-ON (Supplementary Tables 2, 3).

PNS also induced significant changes in the vascular markers ΔSBP, ΔDBP, and ΔMAP as depicted in Figures 4A–C and Supplementary Table 1. The responses show tMNS and taVNS similarly reducing blood pressure more than sham through all timepoints analyzed. tcVNS responses, on the other hand, were more similar to and often higher than those observed for sham. Specifically for ΔSBP, the *post-hoc* tests showed that tMNS significantly reduced the response compared to tcVNS in MA-BRK (Supplementary Tables 2, 3). A significant taVNS-induced reduction in ΔDBP compared to tcVNS was also revealed in MA-STM+STR (Supplementary Tables 2, 3). While ΔPP did not seem to follow a particular trend (Figure 4D), the *post-hoc* tests revealed a significant tMNS-induced reduction of 3.3 mmHg compared to sham in MA-BRK. Further, a significant reduction compared to tcVNS was found in the *post-hoc* tests for MA-BRK (Supplementary Tables 2, 3).

Significant PNS-induced changes were also found in ΔtonicMean, where taVNS and tMNS similarly lowered the response compared to sham for all timepoints after MA-BRK, while tcVNS was found to increase the response compared to sham at most timepoints (Figure 5 and Supplementary Table 1). Specifically, the *post-hoc* tests revealed that tMNS significantly reduced ΔtonicMean by 0.5 and 0.8% in NB-BRK compared to sham and tcVNS, respectively (Supplementary Tables 2, 3). Meanwhile, taVNS was also found to significantly reduce ΔtonicMean by 0.9% compared to tcVNS in NB-BRK (Supplementary Tables 2, 3). A significant stimulation effect was also found in ΔnOR at CP-BRK, MA-BRK, NB-STM-ON, and CP-STM+STR (Supplementary Table 1). Specifically, the *post-hoc* tests revealed that tMNS reduced delta nOR by 2.1 and 1.4% compared to taVNS in MA-BRK and CP-STR+STR, respectively (Supplementary Tables 2, 3).

Finally, taVNS increased ΔPEP and tMNS increased ΔPPGAmp more than the rest of the stimulation types for several timepoints, but the differences were not significant (Supplementary Figures 3B, C and Supplementary Table 3). No significant PNS-induced effects were found for the remaining markers.

### 3.4. Side effects of stimulation

Some participants expressed a general sensation of discomfort or otherwise unpleasant feeling during the calibration period when the stimulation ramp-up reached a value above their final 150% PT. In those cases, the concerns were addressed by reducing the stimulation intensity. One participant reported feeling dizzy and having headaches the morning following the first study visit, in which taVNS was administered. However, it is uncertain whether these effects were taVNS-induced or related to coincidental external personal or environmental factors. The reported side effects disappeared within a day and the subject's participation in the study was terminated to minimize risk.

## 4. Discussion

This single-blind sham-controlled crossover study quantified and compared the acute stress mitigation effectiveness of three prominent PNS modalities (taVNS, tcVNS, tMNS) via the study of physiological signals peripherally measured on 19 young healthy volunteers. The stress responses of a comprehensive set of cardiovascular and sudomotor physiomarkers derived thereof demonstrated that tMNS and taVNS induced changes in the directions of reduced stress. Importantly, these results were not significantly influenced by placebo effects, as revealed by the blinding survey results. These results support further investigation into the use of tMNS and/or taVNS in conjunction with the physiological signals employed herein for novel unobtrusive stress detection and mitigation paradigms.

### 4.1. taVNS and tMNS seem to reduce autonomically-mediated effects of stress

Compared to sham, we found that taVNS and tMNS counteracted cardiac timing effects associated with stress. In particular, taVNS and tMNS increased ΔPEP/LVET and ΔPEP throughout the study with statistically significant increases in ΔPEP/LVET. Similarly, taVNS and tMNS resulted in significant ΔLVETI shortening. These cardiac timing changes suggest reductions in stroke volume (SV), counteracting stress-induced increases in SV. Specifically, LVETI (i.e., HR-corrected LVET) exhibits a direct relationship with SV (Lewis et al., 1977; Atterhög et al., 1981; Hassan and Turner, 1983; Montysaari et al., 1984). Meanwhile, PEP and PEP/LVET have been suggested as prominent makers of cardiac contractility, thereby inversely related to SV through the Frank Starling mechanism (Frey and Siervogel, 1983; Hassan and Turner, 1983; Gurel et al., 2020a).

tMNS relative to sham also induced changes in directions suggesting a reduction in the manifestations of stress in vascular and sudomotor physiomarkers. Notably, tMNS significantly reduced ΔtonicMean, which is a marker of sudomotor activity that is known to be mainly sympathetically-mediated (Giannakakis et al., 2019; Ihmig et al., 2020). tMNS also induced a significant reduction in ΔPP, thereby suggesting a reduction in vascular tone. The reductions in ΔDBP, ΔSBP, and ΔMAP, and the increases in ΔPPGAmp physiomarker responses compared to sham seem to support this speculation, but the changes were not significant.

The lack of significant changes in other physiomarkers points, perhaps, to the intricate relationship between them and the physiological and neurobiological phenomena they aim to quantify. Prior work found tcVNS to reduce physiological manifestations of traumatic stress and opioid withdrawal (Gurel et al., 2020a,b; Gazi et al., 2022a). However, in this study of young healthy adults, we did not find significant effects. This could be at least in part attributed to changes in underlying neurophysiology and autonomic tone in diseased populations. Ultimately, these results highlight the value of the comprehensive set of multimodal physiomarkers employed herein and evidence the need for advanced multimodal and multivariate approaches that can improve the characterization of the complex PNS-induced effects on stress physiology, and comparison studies between healthy and diseased populations.

### 4.2. Implications for continuous unobtrusive wearable-based stress detection and mitigation

While the mental and physiological interventions of this study do not recreate real-life stressors, the individual and aggregated responses they elicit may resemble general classes of daily-life stressors (Allen et al., 1987; Giannakakis et al., 2019). This is important considering that our work evaluated wearable-compatible PNS targets and sensing modalities. Notably, every cardiovascular signal employed herein is compatible with wearables previously studied by our group in a wide range of settings and populations (Chan et al., 2021; Kimball et al., 2021; Semiz et al., 2021; Ganti et al., 2022; Soliman et al., 2022), including BP (Ganti et al., 2021). The cross-integration of SCG and ECG utilized in this work, in particular, enabled the calculation of important stress physiomakers (e.g., ΔPEP/LVET and ΔLVETI) that chiefly captured the PNS-induced effects. Although these makers have been widely-studied in other contexts, their measurement has traditionally required an additional sensing modality that is either obtrusive (impedance cardiography) or requires specialized training (echocardiography; Dehkordi et al., 2019). Regarding stimulation, both taVNS and tMNS naturally lend themselves to ear and wrist-based wearable form-factors, respectively, as demonstrated in recent literature (Bang et al., 2018; Yu et al., 2022). A significant implication of this wearable-amenability is the development of technologies that transition from sporadic intervention to personalized around-the-clock stress detection and mitigation. Furthermore, the physiological signals, physiomarkers, and PNS modalities herein studied may have potential applications in cardiovascular diseases as well, with recent reports of the effective use of taVNS for improving quality of life in patients with atrial fibrillation and heart failure (Stavrakis et al., 2020, 2022).

### 4.3. Limitations and future work

Several limitations of this work should be noted. The study cohort consisted of a limited sample size of young healthy adults. The crossover study protocol employed herein may have been prone to habituation of the participants to the stressors. The sample size was further limited by the multi-visit requirement, leading to several dropouts. The nonuniform distribution of stimulation allocations may have underpowered the statistical analysis and limited the discovery of pure stimulation effects. Likewise, the lack of stimulation-only data for tcVNS (see Section 2.2) may have limited its effects. While the physiomarker responses suggest that the stressors effectively increased the stress levels, it could not be verified that such levels were sufficient for all three PNS modalities to have an effect. Further, in the interest of measuring the effects of various stimulation intensities (e.g., 150% PT) without compromising safety, the final PTs may be below maximal tolerance. Although the blinding analysis results suggest that blinding of the participants was successfully established, this study of four different electrical stimulation anatomical locations over multiple visits may have affected the subjects' perception of active and sham sites due to the likely differences in sensation for each site. While effort was placed in maintaining consistency in the study sessions' scheduled day and time, this was not always possible, and thus the responses may have been influenced by changes in circadian rhythm and mood states. Similarly, although the experiments took place in controlled laboratory conditions, the responses may have been influenced by temperature differences between sessions, inside or outside (e.g., seasonal variations) the experimental site.

Future work will recruit larger and more heterogeneous populations to assess the generalizability of the results obtained herein. To minimize bias and increase the external validity of the results, future studies will explore feasible double-blind procedures. The relationship between varying stimulation intensities and the physiological responses during the dose finding period should also be explored in future work. Further, the correlation between various baseline measures of autonomic tone and the simulation responses may be studied to elucidate the factors influencing the different subject-specific responses. Finally, advanced multivariate approaches will be studied to better infer autonomic nervous system state during stress from physiological signals peripherally measured.

## 5. Conclusion

In this novel single-blind sham-controlled crossover study, we evaluated the stress mitigation effectiveness of three prominent wearable-compatible PNS modalities (i.e., taVNS, tMNS, tcVNS) utilizing a comprehensive set of physiomarkers derived from cardiac, vascular, and sudomotor physiological signals. We have demonstrated that taVNS and tMNS reduced acute stress responses compared to sham in a population of young healthy volunteers. The results presented herein may provide the basis for the development of novel wearable-based neuromodulation paradigms. Such technologies may be particularly beneficial to individuals who suffer from bouts of acute stress. Novel unobtrusive stress management technologies may detect these unsafe increases in stress and correspondingly administer neuromodulation to mitigate it, thereby improving these individuals' quality of life.

## Data availability statement

The raw data supporting the conclusions of this article will be made available by the authors, without undue reservation.

## Ethics statement

The studies involving humans were approved by Georgia Institute of Technology Institutional Review Board. The studies were conducted in accordance with the local legislation and institutional requirements. The participants provided their written informed consent to participate in this study.

## Author contributions

AG, RE, AH, J-OH, and OI contributed to the conception and design of the study. JS-P, AG, FR, AS, GS, and SS contributed to data acquisition. JS-P, RE, and CN contributed to hardware design. JS-P, AG, AS, GS, RE, AH, MM, and YC contributed to software design. JS-P organized and curated the data and wrote the first draft of the manuscript. JS-P and TS performed the statistical analysis. AG wrote sections of the manuscript. All authors contributed to manuscript revision, read, and approved the submitted version.
