# Supplementary material for: Transcutaneous auricular Vagus Nerve Stimulation and Median Nerve Stimulation reduce acute stress in young healthy adults: a single-blind sham-controlled crossover study
Source: Front Neurosci. 2023 Sep 7;17:1213982. doi: 10.3389/fnins.2023.1213982 (PMC10512834; doi:10.3389/fnins.2023.1213982)
Supplement: Supplementary file 1 [file Data_Sheet_1.docx]

Supplementary Material

**Transcutaneous Auricular Vagus Nerve Stimulation and Median Nerve Stimulation Reduce Acute Stress in Young Healthy Adults: A Single-blind Sham-controlled Crossover Study**

**Jesus Antonio Sanchez-Perez** ∗**, Asim H. Gazi, Farhan N. Rahman, Alexis Seith, Georgia Saks, Srirakshaa Sundararaj, Rachel Erbrick, Anna
B. Harrison, Christopher J. Nichols, Mihir Modak, Yekanth R. Chalumuri, Teresa K. Snow, Jin-Oh Hahn, and Omer T. Inan**

*** Correspondence:** Corresponding Author: jasp@gatech.edu

# Supplementary Data

**Detailed signal processing and marker extraction**

**Seismocardiogram (SCG):**

SCG signals were filtered and quality assessed before obtaining a "clean" set of beats from which Systolic Time Intervals (STI) were extracted. A semi-automatic version of our prior SCG processing work was implemented in this work [1]. This process involved re-initializing the core peak tracking algorithm utilized therein as needed to ensure correct tracking of the Aortic Opening (AO) and Aortic Closing (AC) SCG fiducial points throughout the recording [2]. First, all segmented SCG beats in each recording, were visualized in the form of a gray-scale two-dimensional (2D) image with the beat duration in one dimension and the beat number in the other. This technique facilitated the visual tracking of peaks (white color) and valleys (black color) in the beats, as well as noisy segments throughout the recording. Then, the SQI threshold was adjusted empirically for each recording by visualizing the beat-by-beat SQI time series against the 2D image of beats. This empirical threshold was used for the first filtering stage described in [1], which removed beats with SQI values below it. The upper and lower thresholds for the second filtering stage, aimed to remedy false rejection of beats, were adjusted for this particular dataset to 0.65 and 0.25, respectively.

Following signal quality filtering, the final "clean" set of beats was created and visualized with the aforementioned 2D image technique. Contiguous segments of beats, defined by a pair of beat indices, were then annotated automatically utilizing the algorithm proposed in [2]. The AO and AC points were defined as the last and first clearly defined peak and valley in the AO and AC complex, respectively. The beat indices delimiting beat segments, the target extrema point (i.e., peak or valley), the searching window to find the extrema point, and the number of beats to annotate at once were all manually entered in a configuration file, whose entries were loaded iteratively until a recording was processed completely. The Pre-ejection Period (PEP) was computed as the time difference between AO and the corresponding indexing ECG R-peak (in seconds) and the Left-Ventricular Ejection Time (LVET) as the time difference between the AC and AO points. The final PEP and LVET feature time series were formed after removing outliers following the two-stage procedure described in [1] with Mean Absolute Deviation (MAD) thresholds also specified in the configuration file. The ratio of PEP to LVET (PEP/LVET) was then computed after outlier removal. Finally, the Pulse Transit Time (PTT) was computed by subtracting PEP from PAT.

Given the established dependency of LVET on HR, an LVET index (LVETI) was computed as $LVETI= \beta\cdot LVET\left( ms \right)+HR(bpm)$ following the general form of the widely-used Weissler's regression equations for HR correction of STIs [3]–[5]. In this work,$\beta$ was set to 1 following a preliminary regression analysis comparing its HR correction effectiveness against that obtained with the corresponding $\beta$ correction coefficients (1.7 for males and 1.6 for females [3]). Such comparison was done upon noting that the LVET correction coefficients were originally obtained from LVET values derived from carotid arterial pulse tracings and are more commonly applied to LVET measurements obtained with various echo-cardiographic methods [3], [6]. While SCG-derived STIs following traditional manual annotation nomenclature have previously shown acceptable estimation errors compared to these gold-standard methods [7], the measurement of LVET from SCG is intrinsically dependent on such nomenclature. In practice, the AO and AC fiducial points definition is commonly adjusted to maximize automatic tracking consistency under diverse motion and noise artifacts. Therefore, existing HR correction equations may not generalize to SCG-derived STIs for all annotation methods. PEP and PEP/LVET are relatively insensitive to HR and were thus not corrected [4]. Pulse Transit Time (PTT) was computed by subtracting PEP from PAT.

**Electrodermal Activity (EDA):**

The EDA signals were first assessed for quality using the methodology described in [8] with valid signal slope and amplitude ranges modified empirically for this dataset (±9 $\mu$S/s and 0.05-100$\mu$S, respectively). Following imputation of the discarded segments through interpolation, the signal was normalized to zero mean and unit-variance prior to decomposition into the tonic, phasic, and sparse components using the *cvxEDA* convex optimizer [9], [10]. The sparse and tonic components were then used to extract the EDA orienting responses (OR) and tonic statistical features, respectively [11], [12] . The OR were identified as the peaks in the sparse component with minimum prominence and peak height of 0.01. The number of OR (nOR) and mean magnitude of OR (mmOR) features were then computed by counting the peaks and averaging their heights, respectively, within a 10-s non-overlapping window rolled over the entire OR sequence [11]. Three features were finally extracted from the EDA tonic component using the same windowing parameters, namely the mean (tonicMean), standard deviation (tonicSD), and normalized first differences (tonicNFD) [12].

# Supplementary Figures and Tables

## Supplementary Figures

**Supplementary Figure 1.** Custom-made SCG sensing hardware. The **(A)** custom accelerometer module packaged the ADXL354 low-noise tri-axial accelerometer (Analog Devices, Wilmington, MA) and was wired to **(B)** a custom Analog Front-End (AFE) that provided approximately 10x amplification in the frequency range 0.2-60~Hz for each axis separately, i.e., Acc_x_, Accy, Acc_z_. The AFE was powered from a 9 VDC source and was interfaced via wired connection to the accelerometer module and the data acquisition system (MP-150, Biopac Systems, Goleta, CA). Polylactic acid 3-dimensional printing filament was used to manufacture device housing for both modules. In this work, the SCG signal was defined as the acceleration in the dorsoventral axis (Acc_z_).

**
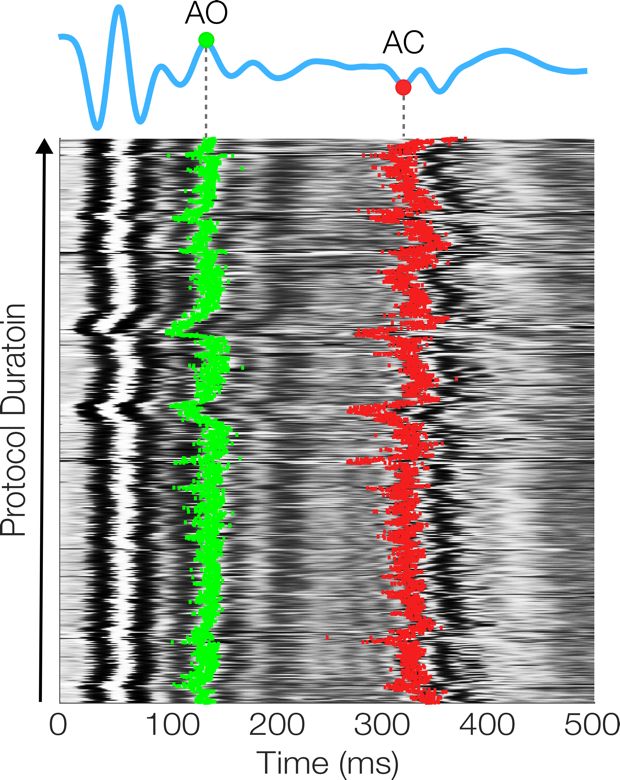
**

**Supplementary Figure 2.** Exemplary SCG annotation following the semi-automatic process used in this work. The resulting AO (green) and AC (red) fiducial points annotations are marked on the ensemble averaged SCG beat (top) and on the 2D beat image stacking the beats for the entire protocol (bottom).

**Supplementary Figure 3.** Physiological responses time series for all stimulation types: **(A)** ∆HR, **(B)** ∆PEP, and **(C)** ∆PPG Amp. Data points are expressed as $\mu\pm SE$.

**Supplementary Table 1** Linear Mixed Effects (LME) models analysis of variance table for the normalized-to-baseline physiomarkers at each time point of interest, with the main visit and stimulation effects and the visit-by-stimulation interaction effects. Significant effects are in bold.

| **Feature** | **Segment** | **Term** | **NumDF** | **DenDF** | **F** | **p** |
| --- | --- | --- | --- | --- | --- | --- |
| DBP | MA STM+STR | visit | 3 | 55 | 2.63 | 0.059 |
|  |  | **stimulation** | **3** | **55** | **3.38** | **0.024** |
|  |  | visit:stimulation | 9 | 63 | 1.41 | 0.205 |
|  | MA BRK | visit | 3 | 55 | 2.49 | 0.070 |
|  |  | **stimulation** | **3** | **55** | **3.02** | **0.037** |
|  |  | visit:stimulation | 9 | 67 | 1.68 | 0.111 |
|  | NB STM+STR | visit | 3 | 54 | 1.84 | 0.151 |
|  |  | **stimulation** | **3** | **54** | **3.18** | **0.031** |
|  |  | visit:stimulation | 9 | 62 | 1.37 | 0.222 |
| SBP | MA STM.ON | visit | 3 | 41 | 1.44 | 0.246 |
|  |  | **stimulation** | **2** | **39** | **3.45** | **0.042** |
|  |  | visit:stimulation | 6 | 51 | 1.38 | 0.242 |
|  | MA BRK | visit | 3 | 55 | 0.26 | 0.857 |
|  |  | **stimulation** | **3** | **55** | **4.31** | **0.008** |
|  |  | visit:stimulation | 9 | 71 | 0.79 | 0.622 |
|  | NB BRK | visit | 3 | 53 | 0.23 | 0.876 |
|  |  | **stimulation** | **3** | **53** | **3.61** | **0.019** |
|  |  | visit:stimulation | 9 | 72 | 0.67 | 0.729 |
| PP | MA BRK | visit | 3 | 53 | 0.97 | 0.414 |
|  |  | **stimulation** | **3** | **53** | **4.98** | **0.004** |
|  |  | visit:stimulation | 9 | 72 | 1.57 | 0.141 |
|  | NB BRK | visit | 3 | 73 | 1.66 | 0.183 |
|  |  | **stimulation** | **3** | **73** | **2.79** | **0.047** |
|  |  | visit:stimulation | 9 | 73 | 1.76 | 0.091 |
| MAP | MA BRK | visit | 3 | 56 | 0.93 | 0.432 |
|  |  | **stimulation** | **3** | **56** | **3.43** | **0.023** |
|  |  | visit:stimulation | 9 | 71 | 1.09 | 0.378 |
|  | NB BRK | visit | 3 | 53 | 0.01 | 0.999 |
|  |  | **stimulation** | **3** | **53** | **4.62** | **0.006** |
|  |  | visit:stimulation | 9 | 69 | 1.08 | 0.387 |
| nOR | CP BRK | visit | 3 | 73 | 2.34 | 0.081 |
|  |  | **stimulation** | **3** | **73** | **4.46** | **0.006** |
|  |  | visit:stimulation | 9 | 73 | 1.72 | 0.099 |
| tonicMean | NB BRK | visit | 3 | 55 | 1.52 | 0.219 |
|  |  | **stimulation** | **3** | **55** | **5.70** | **0.002** |
|  |  | visit:stimulation | 9 | 61 | 1.60 | 0.136 |
|  | CP STM.ON | visit | 3 | 39 | 1.67 | 0.189 |
|  |  | **stimulation** | **2** | **38** | **4.78** | **0.014** |
|  |  | visit:stimulation | 6 | 43 | 1.60 | 0.170 |
| PEP/LVET | MA STM.ON | visit | 3 | 44 | 0.48 | 0.695 |
|  |  | **stimulation** | **2** | **42** | **5.32** | **0.009** |
|  |  | visit:stimulation | 6 | 54 | 1.36 | 0.246 |
|  | NB STM.ON | visit | 3 | 45 | 0.64 | 0.591 |
|  |  | **stimulation** | **2** | **40** | **5.19** | **0.010** |
|  |  | visit:stimulation | 6 | 54 | 1.10 | 0.376 |
|  | NB BRK | visit | 3 | 58 | 0.21 | 0.892 |
|  |  | **stimulation** | **3** | **58** | **3.77** | **0.015** |
|  |  | visit:stimulation | 9 | 71 | 0.89 | 0.542 |
|  | CP STM.ON | visit | 3 | 46 | 0.39 | 0.764 |
|  |  | **stimulation** | **2** | **41** | **4.26** | **0.021** |
|  |  | visit:stimulation | 6 | 54 | 0.76 | 0.605 |
| LVETI | MA STM.ON | visit | 3 | 42 | 0.98 | 0.413 |
|  |  | **stimulation** | **2** | **40** | **6.42** | **0.004** |
|  |  | visit:stimulation | 6 | 54 | 0.99 | 0.440 |
|  | NB STM+STR | visit | 3 | 55 | 1.07 | 0.368 |
|  |  | **stimulation** | **3** | **55** | **4.01** | **0.012** |
|  |  | visit:stimulation | 9 | 66 | 1.85 | 0.075 |
|  | NB STM.ON | visit | 3 | 38 | 1.50 | 0.231 |
|  |  | **stimulation** | **2** | **35** | **7.22** | **0.002** |
|  |  | visit:stimulation | 6 | 53 | 0.70 | 0.652 |
|  | NB BRK | visit | 3 | 73 | 1.52 | 0.216 |
|  |  | **stimulation** | **3** | **73** | **4.96** | **0.004** |
|  |  | visit:stimulation | 9 | 73 | 1.75 | 0.093 |
|  | CP STM.ON | visit | 3 | 41 | 1.63 | 0.198 |
|  |  | **stimulation** | **2** | **38** | **5.26** | **0.010** |
|  |  | visit:stimulation | 6 | 54 | 0.58 | 0.745 |

**Supplementary Table 2** Pairwise post-hoc tests for main stimulation effects at each time point of interest. Significant results (p<0.05) are in bold.

| **Feature** | **Segment** | **Contrast** | **estimate** | **SE** | **df** | **t** | **p** |
| --- | --- | --- | --- | --- | --- | --- | --- |
| DBP | MA STM+STR | sham - tcVNS | -0.28 | 0.31 | 79 | -0.90 | 0.805 |
|  |  | sham - tMNS | 0.12 | 0.31 | 79 | 0.38 | 0.981 |
|  |  | sham - taVNS | 0.47 | 0.30 | 75 | 1.54 | 0.418 |
|  |  | tcVNS - tMNS | 0.40 | 0.27 | 77 | 1.46 | 0.467 |
|  |  | **tcVNS - taVNS** | **0.75** | **0.28** | **78** | **2.68** | **0.044** |
|  |  | tMNS - taVNS | 0.35 | 0.28 | 77 | 1.24 | 0.601 |
| SBP | MA BRK | sham - tcVNS | -0.22 | 0.39 | 81 | -0.58 | 0.939 |
|  |  | sham - tMNS | 0.75 | 0.39 | 81 | 1.91 | 0.233 |
|  |  | sham - taVNS | 0.51 | 0.39 | 75 | 1.32 | 0.553 |
|  |  | **tcVNS - tMNS** | **0.97** | **0.35** | **77** | **2.81** | **0.031** |
|  |  | tcVNS - taVNS | 0.74 | 0.35 | 79 | 2.09 | 0.166 |
|  |  | tMNS - taVNS | -0.24 | 0.36 | 78 | -0.67 | 0.909 |
| PP |  | sham - tcVNS | 0.10 | 0.40 | 81 | 0.26 | 0.994 |
|  |  | **sham - tMNS** | **1.12** | **0.40** | **81** | **2.80** | **0.032** |
|  |  | sham - taVNS | 0.61 | 0.40 | 74 | 1.53 | 0.425 |
|  |  | **tcVNS - tMNS** | **1.02** | **0.36** | **76** | **2.86** | **0.027** |
|  |  | tcVNS - taVNS | 0.51 | 0.36 | 79 | 1.41 | 0.497 |
|  |  | tMNS - taVNS | -0.51 | 0.37 | 78 | -1.39 | 0.506 |
| tonicMean | NB BRK | sham - tcVNS | -0.16 | 0.28 | 78 | -0.57 | 0.942 |
|  |  | sham - tMNS | 0.57 | 0.29 | 78 | 2.00 | 0.197 |
|  |  | sham - taVNS | 0.52 | 0.28 | 75 | 1.90 | 0.239 |
|  |  | **tcVNS - tMNS** | **0.73** | **0.25** | **76** | **2.94** | **0.022** |
|  |  | **tcVNS - taVNS** | **0.68** | **0.25** | **77** | **2.69** | **0.043** |
|  |  | tMNS - taVNS | -0.05 | 0.26 | 76 | -0.18 | 0.998 |
|  | CP STM.ON | **sham - tMNS** | **0.60** | **0.25** | **56** | **2.42** | **0.049** |
|  |  | sham - taVNS | 0.52 | 0.23 | 52 | 2.21 | 0.078 |
|  |  | tMNS - taVNS | -0.08 | 0.22 | 54 | -0.38 | 0.925 |
| PEP/LVET | MA STM.ON | sham - tMNS | -0.83 | 0.36 | 58 | -2.33 | 0.059 |
|  |  | **sham - taVNS** | **-0.92** | **0.35** | **51** | **-2.64** | **0.029** |
|  |  | tMNS - taVNS | -0.09 | 0.32 | 55 | -0.27 | 0.961 |
|  | NB STM.ON | sham - tMNS | -0.93 | 0.40 | 58 | -2.36 | 0.056 |
|  |  | **sham - taVNS** | **-1.03** | **0.40** | **51** | **-2.61** | **0.031** |
|  |  | tMNS - taVNS | -0.10 | 0.36 | 54 | -0.28 | 0.959 |
|  | CP STM.ON | **sham - tMNS** | **-1.01** | **0.41** | **58** | **-2.46** | **0.044** |
|  |  | sham - taVNS | -0.81 | 0.41 | 51 | -1.97 | 0.131 |
|  |  | tMNS - taVNS | 0.20 | 0.37 | 54 | 0.54 | 0.852 |
| LVETI | MA STM.ON | **sham - tMNS** | **1.00** | **0.36** | **59** | **2.80** | **0.019** |
|  |  | **sham - taVNS** | **0.94** | **0.35** | **52** | **2.68** | **0.026** |
|  |  | tMNS - taVNS | -0.06 | 0.32 | 55 | -0.20 | 0.979 |
|  | NB STM.ON | **sham - tMNS** | **0.98** | **0.34** | **61** | **2.88** | **0.015** |
|  |  | **sham - taVNS** | **0.96** | **0.33** | **54** | **2.88** | **0.015** |
|  |  | tMNS - taVNS | -0.02 | 0.31 | 58 | -0.07 | 0.997 |
|  | NB BRK | sham - tcVNS | 0.69 | 0.39 | 80 | 1.79 | 0.287 |
|  |  | **sham - tMNS** | **1.30** | **0.39** | **80** | **3.32** | **0.007** |
|  |  | sham - taVNS | 0.90 | 0.39 | 73 | 2.28 | 0.113 |
|  |  | tcVNS - tMNS | 0.61 | 0.35 | 74 | 1.74 | 0.310 |
|  |  | tcVNS - taVNS | 0.20 | 0.35 | 78 | 0.57 | 0.940 |
|  |  | tMNS - taVNS | -0.41 | 0.36 | 77 | -1.13 | 0.670 |
|  | CP STM.ON | **sham - tMNS** | **1.02** | **0.37** | **59** | **2.77** | **0.020** |
|  |  | sham - taVNS | 0.67 | 0.36 | 52 | 1.86 | 0.161 |
|  |  | tMNS - taVNS | -0.35 | 0.33 | 56 | -1.05 | 0.550 |

**Supplementary Table 3** Physiological markers’ mean and standard deviations for all segments. Stimulation pairs revealed as significantly different during post-hoc statistical procedures are shown in bold.

| **Feat.** | **PNS** | **MA** | | | | | | | **NB** | | | | **CP** | | |
| --- | --- | --- | --- | --- | --- | --- | --- | --- | --- | --- | --- | --- | --- | --- | --- |
|  |  | **STM+STR** | | **STM.ON** | | **BRK** | | | **STM+STR** | | **STM.ON** | **BRK** | **STM+STR** | **STM.ON** | **BRK** |
| HR | sham | 1.3 (8.3) | | -2.7 (5.6) | | -0.9 (5.9) | | | -0.2 (5.1) | | -2.3 (6.7) | -0.9 (5.3) | 4.8 (10.4) | -4.5 (5.9) | -3.3 (6.4) |
|  | tcVNS | 0.7 (6.4) | |  | | 0.0 (6.8) | | | 0.4 (4.9) | |  | -1.7 (5.9) | 6.9 (13.2) |  | -2.6 (6.7) |
|  | tMNS | 1.3 (5.2) | | -0.2 (5.1) | | 1.8 (4.9) | | | -1.0 (5.8) | | -0.6 (5.0) | 0.5 (4.3) | 4.9 (7.2) | -2.9 (4.6) | -0.9 (5.2) |
|  | taVNS | 2.5 (6.0) | | -0.1 (5.5) | | 1.3 (5.6) | | | 0.0 (6.4) | | -0.7 (5.3) | 0.9 (5.3) | 9.3 (12.9) | -2.4 (5.0) | -1.0 (4.4) |
| DBP | sham | 13.8 (7.5) | | 7.6 (6.6) | | 8.2 (7.0) | | | 15.0 (7.5) | | 11.1 (6.9) | 11.7 (6.7) | 28.7 (17.1) | 16.9 (12.1) | 15.2 (11.5) |
|  | tcVNS | **14.9 (4.7)** | |  | | 9.7 (5.5) | | | 15.3 (4.5) | |  | 10.6 (5.0) | 29.3 (11.6) |  | 15.7 (7.7) |
|  | tMNS | 11.9 (6.1) | | 6.7 (6.1) | | 6.1 (5.3) | | | 11.5 (4.9) | | 7.4 (6.3) | 6.9 (5.1) | 24.6 (11.2) | 12.4 (6.3) | 11.7 (5.4) |
|  | taVNS | **12.4 (6.5)** | | 6.4 (4.8) | | 7.2 (5.0) | | | 13.7 (7.4) | | 8.2 (6.3) | 8.9 (5.3) | 26.6 (12.9) | 14.4 (7.4) | 12.1 (6.5) |
| SBP | sham | 17.8 (11.2) | | 12.5 (10.3) | | 13.4 (11.3) | | | 20.1 (12.2) | | 16.7 (11.7) | 18.2 (12.2) | 33.0 (22.1) | 23.2 (19.1) | 22.8 (18.3) |
|  | tcVNS | 20.5 (9.3) | |  | | **15.4 (9.0)** | | | 20.8 (9.0) | |  | 17.6 (7.6) | 33.0 (12.8) |  | 21.2 (10.8) |
|  | tMNS | 16.4 (11.8) | | 10.2 (10.9) | | **8.0 (7.3)** | | | 16.7 (10.0) | | 12.2 (9.1) | 11.9 (8.1) | 31.3 (12.1) | 17.4 (10.7) | 17.1 (10.0) |
|  | taVNS | 15.4 (10.1) | | 9.6 (6.9) | | 10.2 (9.1) | | | 18.5 (10.8) | | 12.0 (10.8) | 12.7 (9.4) | 27.8 (16.2) | 18.5 (12.8) | 16.2 (11.7) |
| MAP | sham |  | 15.8 (8.6) | 9.6 (8.0) | 10.0 (8.3) | |  | 18.0 (9.3) | | 13.5 (8.4) | 14.1 (8.4) |  | 32.2 (19.5) | 21.1 (15.0) | 19.0 (14.1) |
|  | tcVNS |  | 17.5 (5.8) |  | 11.9 (6.2) | |  | 18.2 (5.9) | |  | 13.8 (5.4) |  | 32.3 (12.5) |  | 19.4 (9.2) |
|  | tMNS |  | 14.5 (8.9) | 8.1 (7.9) | 6.7 (5.9) | |  | 14.5 (6.6) | | 9.2 (6.8) | 8.6 (5.3) |  | 28.7 (11.8) | 15.7 (7.5) | 14.3 (6.4) |
|  | taVNS |  | 13.8 (7.8) | 7.6 (4.8) | 7.9 (5.8) | |  | 16.3 (7.9) | | 9.7 (7.1) | 9.9 (5.7) |  | 28.1 (13.8) | 17.3 (9.2) | 14.1 (7.7) |
| PP | sham | 4.0 (5.8) | | 4.9 (5.3) | | **5.2 (5.7)** | | | 5.2 (6.5) | | 5.6 (6.0) | 6.5 (6.8) | 4.3 (7.1) | 6.3 (8.4) | 7.6 (8.7) |
|  | tcVNS | 5.6 (6.3) | |  | | **5.7 (5.9)** | | | 5.5 (5.9) | |  | 7.0 (5.8) | 3.7 (6.0) |  | 5.4 (4.7) |
|  | tMNS | 4.5 (8.0) | | 3.5 (7.7) | | **1.9 (6.5)** | | | 5.2 (8.5) | | 4.9 (7.3) | 5.0 (8.2) | 6.7 (7.1) | 4.9 (8.8) | 5.4 (9.0) |
|  | taVNS | 3.0 (5.3) | | 3.1 (3.7) | | 3.0 (5.3) | | | 4.9 (5.8) | | 3.8 (5.8) | 3.8 (5.5) | 1.2 (8.0) | 4.1 (7.0) | 4.1 (7.2) |
| PPGAmp | sham | -48.1 (34.5) | | -23.7 (48.2) | | -45.1 (23.6) | | | -55.2 (29.1) | | -42.6 (35.5) | -57.5 (23.1) | -78.1 (12.5) | -54.0 (41.1) | -63.8 (22.2) |
|  | tcVNS | -57.0 (29.5) | |  | | -49.0 (23.0) | | | -62.6 (26.6) | |  | -60.1 (20.0) | -80.6 (19.4) |  | -62.8 (28.3) |
|  | tMNS | -53.5 (21.3) | | -25.8 (40.0) | | -37.2 (42.7) | | | -50.1 (26.8) | | -36.2 (36.9) | -46.8 (34.1) | -78.7 (9.9) | -55.2 (23.4) | -64.0 (15.9) |
|  | taVNS | -55.0 (29.1) | | -30.3 (34.9) | | -44.4 (33.7) | | | -62.8 (16.6) | | -40.8 (43.0) | -54.1 (21.8) | -81.2 (12.1) | -59.4 (26.6) | -61.0 (25.7) |
| tonicMean | sham | 1.1 (1.2) | | 0.9 (1.0) | | 0.6 (0.9) | | | 1.5 (1.3) | | 1.3 (1.4) | **1.0 (1.2)** | 1.6 (1.7) | 1.1 (1.2) | 0.5 (1.1) |
|  | tcVNS | 1.3 (1.0) | |  | | 1.1 (1.2) | | | 1.6 (1.0) | |  | **1.3 (1.2)** | 1.4 (1.3) |  | 0.8 (1.3) |
|  | tMNS | 1.4 (1.2) | | 0.9 (1.2) | | 0.5 (1.4) | | | 1.5 (1.3) | | 1.0 (1.3) | **0.5 (1.3)** | 1.2 (1.3) | 0.6 (1.2) | -0.0 (1.2) |
|  | taVNS | 1.2 (1.2) | | 0.9 (1.1) | | 0.5 (1.2) | | | 1.4 (1.3) | | 1.0 (1.1) | **0.4 (1.0)** | 1.4 (1.5) | 0.7 (1.2) | 0.1 (1.1) |
| PEP | sham | -3.2 (5.7) | | -1.4 (4.8) | | -0.4 (6.0) | | | -5.0 (5.1) | | -1.1 (4.3) | -1.1 (4.8) | -9.2 (9.8) | -3.2 (5.1) | -1.3 (4.7) |
|  | tcVNS | -3.6 (7.4) | |  | | -1.2 (5.5) | | | -3.1 (6.2) | |  | -1.4 (5.8) | -8.7 (8.4) |  | -2.8 (5.9) |
|  | tMNS | -3.9 (7.6) | | -0.1 (5.7) | | 0.1 (6.5) | | | -3.0 (6.0) | | -0.0 (4.4) | 0.4 (4.9) | -8.7 (8.7) | -1.2 (4.9) | -1.7 (6.1) |
|  | taVNS | -2.3 (6.8) | | 1.8 (4.8) | | 1.4 (4.4) | | | -2.1 (6.2) | | 1.5 (4.5) | 2.1 (5.0) | -7.6 (9.2) | -0.9 (5.4) | -0.0 (6.4) |
| LVETI | sham | 2.6 (2.8) | | **2.6 (2.8)** | | 2.7 (2.6) | | | 4.4 (3.5) | | **3.4 (2.6)** | **3.2 (3.0)** | 5.6 (4.2) | **5.3 (3.0)** | 2.8 (2.8) |
|  | tcVNS | 2.5 (3.4) | |  | | 1.3 (3.6) | | | 3.2 (3.4) | |  | 2.2 (2.6) | 5.6 (4.3) |  | 3.9 (3.4) |
|  | tMNS | 1.7 (2.1) | | **0.7 (1.9)** | | 0.3 (2.3) | | | 2.0 (2.5) | | **1.2 (2.5)** | **0.5 (2.0)** | 5.0 (2.8) | **2.1 (4.0)** | 1.9 (2.9) |
|  | taVNS | 1.5 (3.1) | | **1.1 (2.4)** | | 1.3 (2.5) | | | 2.6 (2.4) | | **1.3 (2.4)** | 1.3 (2.4) | 4.8 (5.0) | 3.6 (3.8) | 2.7 (2.9) |
| PEP/LVET | sham | -5.4 (7.4) | | **-5.3 (7.7)** | | -3.7 (8.5) | | | -9.8 (7.2) | | **-5.9 (5.8)** | -5.0 (6.7) | -13.3 (11.5) | **-10.8 (6.4)** | -5.5 (8.2) |
|  | tcVNS | -6.0 (10.0) | |  | | -2.3 (9.6) | | | -6.5 (9.6) | |  | -4.5 (8.9) | -12.0 (11.0) |  | -8.1 (9.1) |
|  | tMNS | -5.3 (9.2) | | -0.8 (6.8) | | 1.0 (9.0) | | | -5.6 (8.0) | | -1.5 (5.8) | 0.2 (6.4) | -12.2 (10.5) | -4.8 (6.7) | -4.1 (8.4) |
|  | taVNS | -3.0 (8.7) | | **0.6 (7.5)** | | 0.3 (6.3) | | | -5.1 (8.4) | | **-0.4 (5.9)** | 1.1 (7.2) | -9.2 (12.0) | **-6.1 (7.9)** | -3.7 (7.8) |
|  |  |  | |  | |  | | |  | |  |  |  |  |  |

**Supplementary Table 4** Blinding survey contingency tables for each visit.

|  | **Actual Stimulation Type** | | | | | | | |
| --- | --- | --- | --- | --- | --- | --- | --- | --- |
| **Surveyed Stim.** | **Visit 1** | | | | **Visit 2** | | | |
|  | **sham** | **tcVNS** | **tMNS** | **taVNS** | **sham** | **tcVNS** | **tMNS** | **taVNS** |
| sham | 1 | 1 | 2 | 1 | 1 | 1 | 1 | 0 |
| tcVNS | 1 | 1 | 0 | 2 | 0 | 0 | 0 | 2 |
| tMNS | 0 | 0 | 3 | 1 | 0 | 2 | 1 | 2 |
| taVNS | 2 | 1 | 2 | 1 | 0 | 4 | 1 | 4 |
| Total | 4 | 3 | 7 | 5 | 1 | 7 | 3 | 8 |
| **Surveyed Stim.** | **Visit 3** | | | | **Visit 4** | | | |
|  | **sham** | **tcVNS** | **tMNS** | **taVNS** | **sham** | **tcVNS** | **tMNS** | **taVNS** |
| sham | 1 | 2 | 0 | 2 | 1 | 1 | 3 | 0 |
| tcVNS | 3 | 1 | 0 | 0 | 2 | 0 | 0 | 1 |
| tMNS | 3 | 1 | 1 | 0 | 0 | 2 | 3 | 1 |
| taVNS | 3 | 0 | 1 | 1 | 1 | 0 | 1 | 0 |
| Total | 10 | 4 | 2 | 3 | 4 | 3 | 7 | 2 |

**Supplementary Table 5** Chi-square of independence test table between actual and surveyed stimulation types

| **Visit** | **DF** | $\boldsymbol{\chi}^{\boldsymbol{2}}$ | **p** |
| --- | --- | --- | --- |
| 1 | 9 | 10.19 | 0.695 |
| 2 | 9 | 6.44 | 0.335 |
| 3 | 9 | 8.57 | 0.478 |
| 4 | 9 | 9.44 | 0.398 |

**References**

[1] A. H. Gazi *et al.*, “Transcutaneous Cervical Vagus Nerve Stimulation Inhibits the Reciprocal of the Pulse Transit Time’s Responses to Traumatic Stress in Posttraumatic Stress Disorder ^*^,” in *2021 43rd Annual International Conference of the IEEE Engineering in Medicine & Biology Society (EMBC)*, Mexico, Nov. 2021, pp. 1444–1447. doi: 10.1109/EMBC46164.2021.9630415.

[2] J. Zia, J. Kimball, M. H. Shandhi, and O. T. Inan, “Automated Identification of Persistent Time-Domain Features in Seismocardiogram Signals,” in *2019 IEEE EMBS International Conference on Biomedical & Health Informatics (BHI)*, Chicago, IL, USA, May 2019, pp. 1–4. doi: 10.1109/BHI.2019.8834555.

[3] A. M. Weissler, W. S. Harris, and C. D. Schoenfeld, “Systolic Time Intervals in Heart Failure in Man,” *Circulation*, vol. 37, no. 2, pp. 149–159, Feb. 1968, doi: 10.1161/01.CIR.37.2.149.

[4] S. Hassan and P. Turner, “Systolic time intervals: a review of the method in the non-invasive investigation of cardiac function in health, disease and clinical pharmacology.,” *Postgraduate Medical Journal*, vol. 59, no. 693, pp. 423–434, Jul. 1983, doi: 10.1136/pgmj.59.693.423.

[5] M. Montysaari, K. Antila, and T. Peltonen, “Relationship between systolic time intervals and heart rate during four circulatory stress tests,” *Europ. J. Appl. Physiol.*, vol. 52, no. 3, pp. 282–286, Sep. 1984, doi: 10.1007/BF01015210.

[6] A. S. Alhakak, J. R. Teerlink, J. Lindenfeld, M. Böhm, G. M. C. Rosano, and T. Biering‐Sørensen, “The significance of left ventricular ejection time in heart failure with reduced ejection fraction,” *European J of Heart Fail*, vol. 23, no. 4, pp. 541–551, Apr. 2021, doi: 10.1002/ejhf.2125.

[7] P. Dehkordi *et al.*, “Comparison of Different Methods for Estimating Cardiac Timings: A Comprehensive Multimodal Echocardiography Investigation,” *Front. Physiol.*, vol. 10, p. 1057, Aug. 2019, doi: 10.3389/fphys.2019.01057.

[8] I. R. Kleckner *et al.*, “Simple, Transparent, and Flexible Automated Quality Assessment Procedures for Ambulatory Electrodermal Activity Data,” *IEEE Trans Biomed Eng*, vol. 65, no. 7, pp. 1460–1467, Jul. 2018, doi: 10.1109/TBME.2017.2758643.

[9] A. Greco, G. Valenza, A. Lanata, E. Scilingo, and L. Citi, “cvxEDA: a Convex Optimization Approach to Electrodermal Activity Processing,” *IEEE Trans. Biomed. Eng.*, pp. 1–1, 2016, doi: 10.1109/TBME.2015.2474131.

[10] A. Greco, A. Lanata, L. Citi, N. Vanello, G. Valenza, and E. Scilingo, “Skin Admittance Measurement for Emotion Recognition: A Study over Frequency Sweep,” *Electronics*, vol. 5, no. 4, p. 46, Aug. 2016, doi: 10.3390/electronics5030046.

[11] A. H. Gazi *et al.*, “Respiratory Markers Significantly Enhance Anxiety Detection Using Multimodal Physiological Sensing,” in *2021 IEEE EMBS International Conference on Biomedical and Health Informatics (BHI)*, Athens, Greece, Jul. 2021, pp. 1–4. doi: 10.1109/BHI50953.2021.9508589.

[12] F. R. Ihmig, A. G. H., F. Neurohr-Parakenings, S. K. Schäfer, J. Lass-Hennemann, and T. Michael, “On-line anxiety level detection from biosignals: Machine learning based on a randomized controlled trial with spider-fearful individuals,” *PLoS ONE*, vol. 15, no. 6, p. e0231517, Jun. 2020, doi: 10.1371/journal.pone.0231517.
